# Extended phase space thermodynamics of regular-AdS black hole

**DOI:** 10.1038/s41598-024-62645-4

**Published:** 2024-06-16

**Authors:** Mohd Rehan, Shafqat Ul Islam, Sushant G. Ghosh

**Affiliations:** 1 Centre for Theoretical Physics, Jamia Millia Islamia, New Delhi, 110025 India; 2 Department of Physics, Jamia Millia Islamia, New Delhi, 110025 India; 3 Astrophysics and Cosmology Research Unit, School of Mathematics, Statistics and Computer Science, University of KwaZulu-Natal, Private Bag 54001, Durban, 4000 South Africa

**Keywords:** Regular-AdS black hole, Thermodynamics, Critical exponents, Astronomy and astrophysics, Astronomy and astrophysics

## Abstract

After obtaining an exact regular-AdS black hole resulting from the coupling of general relativity with nonlinear electrodynamics (NED), we explore the thermodynamics of the extended phase space, treating the cosmological constant ($\Lambda$) as the pressure (*P*) of the black holes and its conjugate as thermodynamic volume (*V*). Considering the NED parameter (*g*), we investigate the Hawking temperature, entropy, Gibb’s free energy and specific heat at the horizon radius. Due to the presence of NED charge, the black hole exhibits van der Waals-like phase transition instead of Hawking-Page phase transition, which could be observed through the $G-T$ plots, which display a swallowtail pattern below the critical pressure, and it gives rise to second-order phase transitions when pressure attains its critical value. The first-order phase transition shares similarities with the liquid-gas phase transition. We determine the exact critical points and explore the influence of NED on $P-V$ criticality, revealing that the isotherms undergo a liquid-gas-like phase transition for temperatures below its critical value $T_C$, especially at lower $T_C$. The identical critical exponent to that of the van der Waals fluid suggests that the NED does not alter the critical exponents, as observed in other arbitrary AdS black holes.

## Introduction

Black holes hold a significant place in astrophysics as beautiful and enigmatic objects. Their existence was initially theorized within the general relativity (GR) framework. However, GR encounters a limitation by predicting singularities at the core of a black hole, failing to account for quantum mechanical effects. The quest for a comprehensive theory of quantum gravity continues as an active area of research, aiming to address and resolve these issues. The investigation of the thermodynamics of black holes has revealed a profound and essential connection between gravity, thermodynamics, and quantum theory. Through classical and semiclassical analysis, this investigation has significantly contributed to our current understanding of quantum phenomena in strong-gravitational forces^1,2^. The development of quantum field theory on curved surfaces has established a relationship between the surface gravity and the temperature of a black hole^3^, as well as the area of its event horizon and entropy^4^. A groundbreaking study by Hawking and Page^5^ discovered a phase transition between Schwarzschild AdS black holes and thermal AdS space, captivating astrophysicists and motivating further examination of black hole thermodynamics in AdS spacetimes. Notably, recent advances have introduced the treatment of a negative cosmological constant as a thermodynamic pressure (*P*) with its corresponding conjugate thermodynamic volume (*V*), modifying the first law of black hole thermodynamics to include a new term, *VdP*. Accordingly, the black hole mass now assumes the role of enthalpy^6–9^, rather than internal energy. Recent investigations into $P-V$ criticality in the extended phase space, developed by Kubizňák et al^10^, have sparked a revolution^11–24^ in black hole phase transition research. Kubizňák et al ,^10^ have elegantly established a complete analogy between charged AdS black holes and the liquid-gas system initially observed by Chamblin et al.^25,26^ The exploration of $P-V$ criticality stems from the growing attention given to the variation of the cosmological constant in the first law of black hole thermodynamics^27–33^, a concept firmly grounded in physical interpretation^10^. A significant shift occurred in understanding the thermodynamics of anti-de Sitter (AdS) black holes when the cosmological constant was perceived as the thermodynamic pressure and its variation was incorporated into the first law^10,19,34–40^. This novel perspective is called black hole chemistry^39,40^.

Our aim here is to establish a connection between regular black holes and the concept of black hole chemistry. We build upon the work by Fan and Wang^41^ by incorporating a negative cosmological constant into the gravitational action and for a particular Lagrangian, which derives a class of exact regular-anti-de Sitter black holes for the purpose. GR encounters a limitation by predicting singularities at the core of a black hole, failing to account for quantum mechanical effects. The quest for a comprehensive theory of quantum gravity continues as an active area of research, aiming to address and resolve these issues. However, we are still working on a specific theory of quantum gravity. Without well-defined quantum gravity, models of regular black holes have received significant attention^42^. The regular black holes, motivated by quantum arguments, are solutions with horizons such that their metrics and curvature invariants are well-behaved everywhere, including at the centre^42^. Regular black holes have gained substantial recognition for their potential in resolving singularity issues. This concept traces back to Bardeen, who introduced the pioneering model of a regular black hole^43^. In this framework, horizons exist while singularities are absent. The study of regular black holes has since made remarkable strides, leading to a significant body of research delving into their properties^42,44–60^, unveiling exciting insights and advancements. Also, the regular black hole^41^ in question is physically reasonable because it has no curvature singularity, the energy-momentum tensor satisfies the standard energy conditions on the event horizon of a large black hole, realized for a non-zero measure set in the black hole solution’s parameter space and also dynamically stable^61^.

We organize the paper as follows: In “Regular black hole”, we derive an exact regular-AdS black hole resulting from the coupling of general relativity with NED^62^ and examine its horizon structure. We also address the energy conditions within the same section. Moving on to “Extended phase space thermodynamics”, we inquire into the extended phase space thermodynamics of the regular-AdS black hole and discuss its stability. The investigation of $P-V$ criticality is presented in “Critical exponents”. Finally, in “Concluding remarks”, we provide concluding remarks for the paper.

## Regular black hole

We consider Einstein’s general relativity with a negative cosmological term coupled to NED given by the action^63,64^.

1 $$\begin{aligned} \textbf{S} = \frac{1}{16\pi }{\int d^4x\sqrt{-g}\Bigg (R+\frac{6}{l^2}-4{\mathscr {L}}({\mathscr {F}})\Bigg )}, \end{aligned}$$

where *R* is the Ricci scalar, *g* is the determinant of the metric tensor and *l* is the positive AdS radius connected with the cosmological constant $\Lambda$ through the relation $\Lambda = -3/{l^2}$. Varying action in Eq. (1) leads to the following field equations of motion

2 $$\begin{aligned} G_{\mu \nu } - \frac{3}{l^2} g_{\mu \nu }= & {} 2\Big [\frac{\partial {\mathscr {L}}({\mathscr {F}})}{\partial {\mathscr {F}}}F_{\mu \lambda } F ^{\lambda }_{\nu } - g_{\mu \nu }{\mathscr {L}}({\mathscr {F}})\Big ], \end{aligned}$$

3 $$\begin{aligned} \nabla _\mu \Big (\frac{\partial \ {\mathscr {L}}({\mathscr {F}})}{\partial {\mathscr {F}}}F^{\nu \mu }\Big )= & {} 0, \end{aligned}$$

4 $$\begin{aligned} \nabla _\mu (*F^{\mu \nu })= & {} 0, \end{aligned}$$

where $G_{\mu \nu }$ is the Einstien tensor. The Lagrangian structure for NED must be uncomplicated and uphold specific physical criteria for regular matter fields. These criteria encompass the weak energy condition (WEC), while the potential strong energy condition (SEC) violation is admissible. Guided by these prerequisites, we derived the NED Lagrangian density ${\mathscr {L}}({\mathscr {F}})$, wherein ${\mathscr {F}} = \frac{1}{4} F^{\mu \nu } F_{\mu \nu }$, and is given by

5 $$\begin{aligned} {\mathscr {L}}({\mathscr {F}}) = \frac{24{\mathscr {F}}}{s(2^\frac{3}{4}+2{\mathscr {F}}^{\frac{1}{4}}\sqrt{q})^4}, \end{aligned}$$

where *s* is a parameter that will be fixed later. To obtain the regular black hole solution, we assume the static and spherically symmetric ansatz as given by

6 $$\begin{aligned} ds^2 = -f(r)dt^2 + f(r)^{-1}dr^2 + r^2(d\theta ^2 + sin^2(\theta )d\phi ^2). \end{aligned}$$

The function *f*(*r*) is to be determined by solving the field Eq. (2). For a spherically symmetric spacetime, the only non-vanishing components for $F_{\mu \nu }$ are $F_{01}$ and $F_{23}$. Further, we consider the Maxwell field tensor as follows

7 $$\begin{aligned} F_{\mu \nu }= 2\delta ^{\theta }_{[\mu }\delta ^{\phi }_{\nu ]}Z(r,\theta ). \end{aligned}$$

With this ansatz substituted in Eq. (3), which may be readily integrated to yield

8 $$\begin{aligned} F_{\mu \nu } = 2\delta ^{\theta }_{[\mu }\delta ^{\phi }_{\nu ]}q(r)\sin {\theta }. \end{aligned}$$

Using Eq. (4), we obtain $q'(r)\sin {\theta }dr\wedge d\theta \wedge d\phi = 0$, which results in the assertion that *q*(*r*) = const = *q*. The magnetic monopole charge is indicated by *q* here^65^. Consequently, the magnetic field strength is given by

9 $$\begin{aligned} F_{23}= 2q\sin {\theta }, \quad and \quad {\mathscr {F}} = \frac{q^2}{2r^4}. \end{aligned}$$

By substituting the value of ${\mathscr {F}}$ from Eq. (9) into Eq. (5), we obtain

10 $$\begin{aligned} {\mathscr {L}}(r) =\frac{3Mq}{(r+q)^4}. \end{aligned}$$

Finally, solving Einstien equation, Eq. (2), using Eq. (6) and Eq.(10), we obtain the solution

11 $$\begin{aligned} f(r) = 1 - \frac{2Mr^2}{(q+r)^3}+\frac{r^2}{l^2}. \end{aligned}$$

where the parameter *s* is related to the magnetic charge and the mass of the black hole via $s=q/2M.$ The Eq. (11) can be identified as a special case of general regular black holes^41,51^ when $\nu =1,\; \mu =3$. Importantly, the regular AdS black hole in Eq. (11) encompasses the well-known Schwarzschild AdS black hole when NED is switched off ($q = 0$).

**Figure 1:**
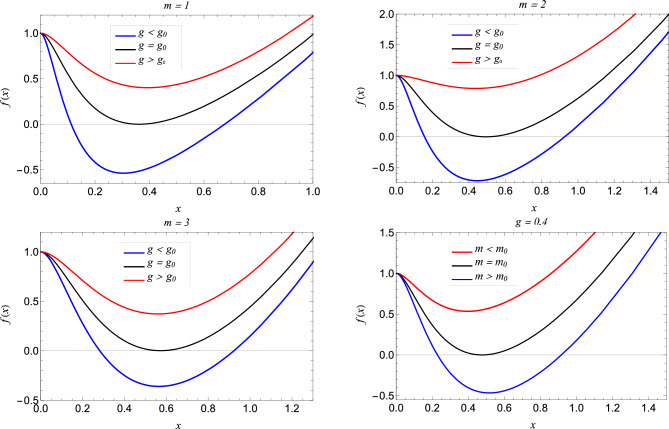
The plots showing metric function *f*(*x*) vs *x* for regular-AdS black holes. The extremal black hole with degenerate horizon occur for $g= g_0$ and $m=m_0$ and the black hole with Cauchy and event horizons exist for $m > m_0$ and $g < g_0$.

Next, we aim to investigate the thermodynamics of black hole  (11), and for this purpose, we rewrite the solution in units of *l* as

12 $$\begin{aligned} f(x) = 1 - \frac{2mx^2}{(g+x)^3} + x^2, \end{aligned}$$

such that $x = r/l$, $m = M/l$, and $g = q/l$. For $x \gg g$, the solution mimics the Schwarzschild AdS, while for $x\rightarrow 0$, the metric function (12) reduces to

13 $$\begin{aligned} f(x) \approx 1+x^2 \Lambda _{\text {eff}} , \;\; \Lambda _{\text {eff}} = \left( 1-\frac{2m}{g^3}\right) . \end{aligned}$$

**Figure 2:**
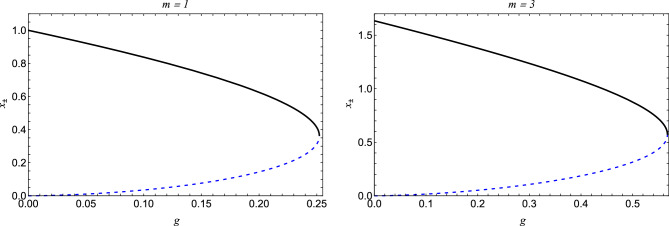
The behaviour of radius coordinate of event horizon (solid lines), radius coordinate of Cauchy horizon (dashed lines) as a function of *g*.

**Figure 3:**
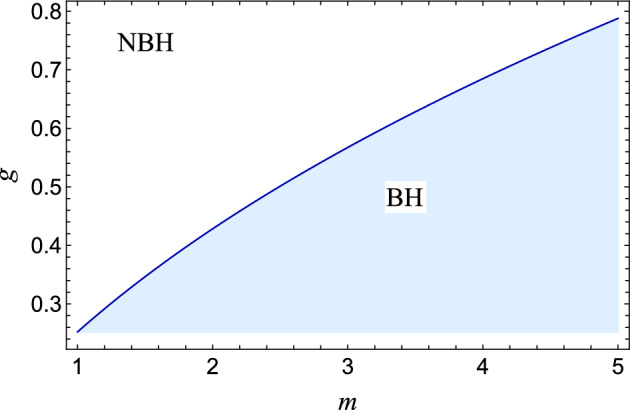
The parameter space for regular-AdS black hole. The dark-blue line corresponds to parameter values for extremal black holes separating black hole (BH) spacetime from no black hole (NBH) spacetime..

We want to study the horizon structure of the black hole. Given a specific *g*, the critical value of *m* can be determined such that for $m > m_0$, two roots ($x_{\pm }$) of $f(x) = 0$ exist, corresponding to the Cauchy horizon $(x_{-})$ and the event horizon $(x_{+})$ respectively. The degeneration of the two horizons occurs at $(x_+ = x_- = x_0)$ when $m = m_0$, leading to extremal black holes. Similarly, a critical value of *g* for a given *m* can be established, where $g = g_0$ corresponds to an extremal black hole, and $g < g_0$ corresponds to regular black holes with two horizons. The variation of event and Cauchy horizons concerning *g* for various *m* values is plotted in (cf. Figs. 1 and 2), revealing that the Cauchy horizon grows as *g* increases, while the event horizon decreases. In the parametric space (*m*, *g*) shown in (cf. Fig. 3), the solid blue curve represents extremal black holes, the blue region depicts black holes with two horizons, and the white region indicates the absence of horizons.

As expected, the solution near the origin becomes an AdS vacuum solution. We compute curvature polynomials to ensure the geometry is regular at the origin and discover that they all have a finite value at $x=0$. In the absence of cosmological constant, the Ricci scalar *R* $= R^{ab}R_{ab}$ and Kretschmann scalar *K* $= R^{abcd}R_{abcd}$ have the following form

14 $$\begin{aligned} R = \frac{24 m g^2}{(x+g)^2}, ~~~~~~~~~ K = \frac{48 m^2(24g^2+7g^2x^2-2gx^3+x^4)}{(x+g)^{10}}. \end{aligned}$$

These invariants exhibit smooth behaviour throughout spacetime, even at $x = 0$. This characteristic underscores the solution’s regularity. This contrasts with classical black holes like the Schwarzschild and Reissner-Nordström black holes, which possess a singularity at the origin.

Next, we check the status of the various energy conditions using the prescription of Hawking Ellis^3^. The Einstein equations governing the stress-energy tensor for the metric in Eq. (12) lead to the following

15 $$\begin{aligned} \rho= & {} \frac{3}{(x+g)^4}\Big [g^4 + 4g^3 x + 6 g^2 x^2 +4 g x^3 + x^4 - 2mg\Big ] = -P_x, \end{aligned}$$

16 $$\begin{aligned} P_\theta= & {} P_\phi = \frac{3}{(x+g)^5}\Big [g^5 + 5g^4 x + 10 g^3 x^2 + 10 g^2 x^3 + 5 g x^4 + x^5 - 2 m g^2 + 2 m g x\Big ]. \end{aligned}$$

**Figure 4:**
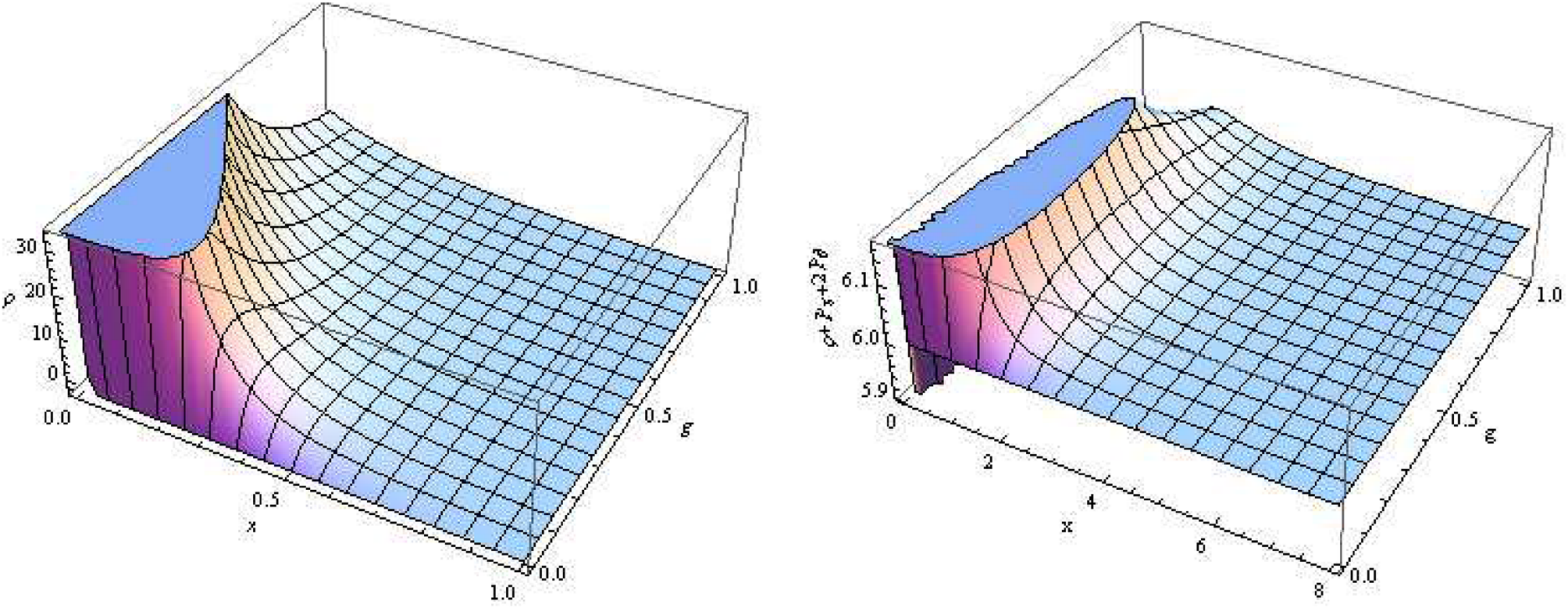
Plot of $\rho$ (left) and (b) $\rho +P_x + 2P_\theta$ (right) vs radius *x* and charge *g*.

The WEC requires $T_{\mu \nu } t^\mu t^\nu \ge 0$ everywhere, for any timelike vector $t^\mu$ which is equivalent to^66^.

17 $$\begin{aligned} \rho \ge 0, \quad \rho +P_i\ge 0 \; (i=1,\;2,\;3), \end{aligned}$$

and hence

18 $$\begin{aligned} \rho +P_\theta = \rho +P_\phi = \frac{12mgx}{(x+g)^5}, \end{aligned}$$

hence the WEC is satisfied (cf. Fig. 4). Next, the NEC requires that $T_{\mu \nu }\ge 0$ in the entire spacetime for any null vector $t_\mu$. Furthermore, the null energy condition demand $\rho +P_x \ge 0$ and $\rho + P_\theta \ge 0$. The former becomes identically zero, and $\rho + P_\theta$ is positive. Hence the NEC is also satisfied. Finally, the SEC states that $T_{\mu \nu }\ge 1/2T^{\mu }_{\nu }t^\nu t^{\mu }$ globally, for any timelike vector $t^{\mu }$, which requires an additional condition, i.e.,

19 $$\begin{aligned} \rho + P_{x}+2 P_{\theta } \ge 0. \end{aligned}$$

Thus, the strong energy criterion is likewise satisfied (cf. Fig. 4).

## Extended phase space thermodynamics

The exploration of black hole thermodynamics is a captivating and intriguing field of research. Next, we search into the thermodynamic properties of regular-AdS black holes. John Wheeler^67,68^ showed that the entropy of any system containing a black hole does not adhere to the non-decreasing entropy principle, making it necessary to assign temperature and entropy values to a black hole. This crucial development prompted Bekenstein and Hawking^3,4,69^ to make a groundbreaking contribution to black hole thermodynamics by relating the temperature and entropy of black holes at the event horizon to the surface gravity and the area of the event horizon. Additionally, the metric function (12) exhibits asymptotic behaviour at infinity, enabling us to construct a conserved mass energy concerning the event horizon. Thus, the analysis of all thermodynamic quantities of the black hole is associated with the horizon $x_{+}$. The black hole mass is obtained from the relation $f(x_+) = 0$ as

20 $$\begin{aligned} m_+ = \frac{(g + x_{+} )^3(1+x_{+}^2)}{2{x_{+}}^2}. \end{aligned}$$

Using Eq. (12), the Hawking temperature (*T*) of the black hole can be derived from the definition of surface gravity $\kappa$^40^ as follows

21 $$\begin{aligned} T_+ = \frac{1}{4{\pi }x_{+}}\Big [\frac{3x_{+}^3 + x_{+}-2g}{(g+x_{+})}\Big ]. \end{aligned}$$

The temperature has to be non-negative, i.e., $3x_+^3 -2g + x_+ \ge 0$, which implies

22 $$\begin{aligned} x_+ \ge \frac{-1+\Big (9g + \sqrt{1 + 81g^2}\Big )^{2/3}}{3\Big (9g +\sqrt{1+81g^2}\Big )^{1/3}}= x_0. \end{aligned}$$

**Figure 5:**
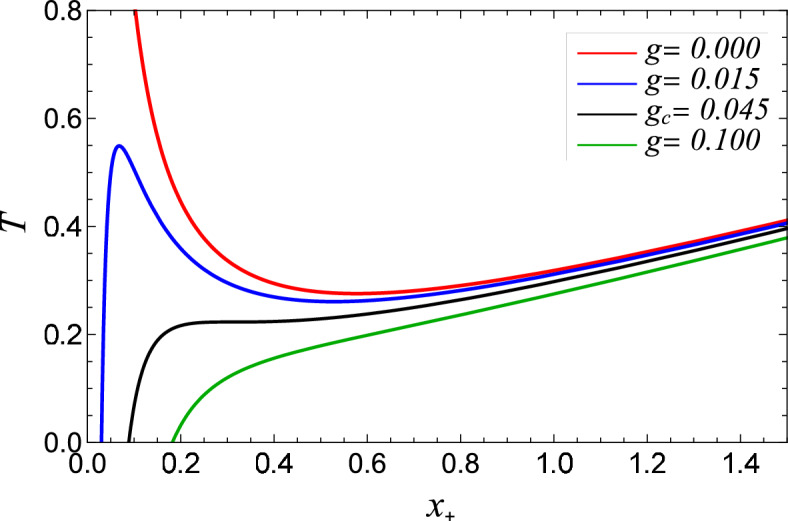
The Hawking temperature $T$ vs the horizon $x_+$ for various values of charge *g*. For $g = 0.015 < g_c$, $T_{max} = 0.54908$ at $x_+ = 0.0679$ and $T_{min} =0.26079$ at $x_+=0.52829$. For $g= g_c= 0.045314421$, $T_{max} =$ $T_{min}= 0.223056$ at $x_+ = 0.311$.

We find the position of the local Hawking temperature extrema in Eq. (21) by solving

23 $$\begin{aligned} \frac{\partial T}{\partial x}\Big |_{x_+=x_0}=\frac{\partial ^2T}{\partial x^2}\Big |_{x_+ = x_o} = 0, \end{aligned}$$

which leads to the critical value of *g* denoted by $g_c = 0.045314421$. When $g < g_c$, the temperature exhibits one maximum and one minimum point, which is shown in the blue curve in Fig. 5. As *g* increases within this range, the temperature initially ascends to a peak value and then descends to a minimum value. At the critical charge $g = g_c$, the two extrema of the temperature curve converge into a single inflexion point, demonstrated by the merging of the blue and black curves in Fig. 5. Beyond this point, for $g > g_c$, the temperature becomes a consistently increasing function concerning the radius, which is indicated by the solid red curve in Fig. 5. As the charge surpasses the critical value, the temperature keeps rising steadily, presenting no extrema or inflexion points as shown by the red curve in Fig. 5.

Further, the black hole’s Bakenstein–Hawking entropy (*S*) is obtained from the first law of black hole thermodynamics. The entropy of the regular AdS black hole is obtained by replacing Eq. (20) and Eq. (21) in first law’s Thermodynamic and integrating from $x_0$, the minimum radius, up to $x_+$, the radius of the horizon, which yields

24 $$\begin{aligned} S = 2{\pi }x^2\Big [\frac{3g}{x}+ \frac{3g^2}{x^2}\log {x} - \frac{g^3}{x^3} + \frac{1}{2} \Big ]\Biggr |_{x_0}^{x_{+}}. \end{aligned}$$

The standard area law $\big (S\approx \pi x^2 = A/4\big )$ is modified due to NED and is obtained in the classical limit ($x_+\gg g$). In the realm of extended phase space thermodynamics, we regard the negative cosmological constant, denoted as $\Lambda$, as equivalent to a thermodynamic pressure and its conjugate volume, denoted as (*P*) and (*V*) repectively, through a transformative process wherein

25 $$\begin{aligned} P = -\frac{\Lambda }{8 \pi } = \frac{3}{8\pi l^2}, \quad V = \frac{4}{3}\pi (g + x)^3. \end{aligned}$$

**Figure 6:**
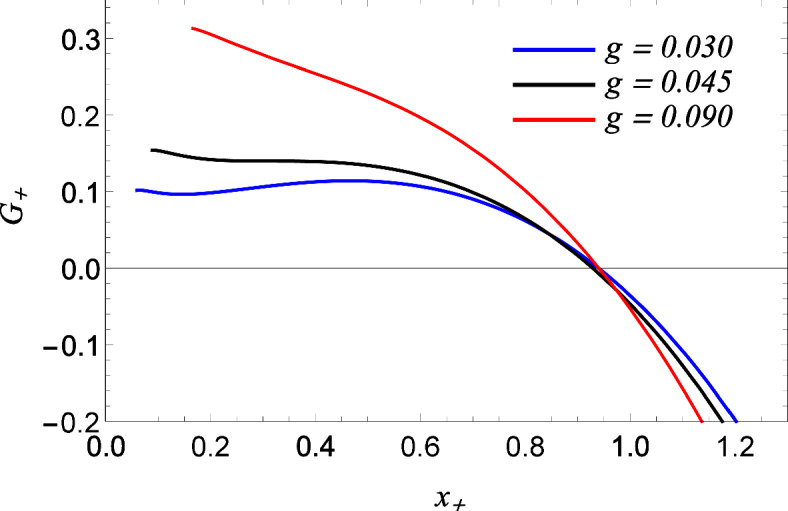
The plot of Gibbs free energy vs $x_+$ for $g = 0.03$ (Blue solid curves), for $g = g_c \approx$ 0.0453 (black curves) and for $g = 0.09$ (Red solid curves).

Moreover, the extended phase space thermodynamics, which treats the black hole mass as enthalpy, provides a more thorough understanding of black hole thermodynamics. In addition, it is possible to consider the black hole mass as a function of three thermodynamic parameters rather than just two, with the relation $m = m(S, P, g)$. In this manner, the contributions from the *g* into the Smarr relation and the first law’s thermodynamics are considered. Because of the additional parameter *g* in the regular AdS black hole, the differential of the mass will give

26 $$\begin{aligned} dm= \Big (\frac{\partial {m}}{\partial {S}}\Big )_{P,g}dS +\Big (\frac{\partial {m}}{\partial {P}}\Big )_{S,g}dP+\Big (\frac{\partial {m}}{\partial {g}}\Big )_{S,P}dg. \end{aligned}$$

with the quantity $\phi$ $=$ $\Big (\frac{\partial {m}}{\partial {g}}\Big )$ being the conjugate variable of *g*. The first law of black hole thermodynamics for a charged and static black hole in extended phase space gets modified by including charge and cosmological constant along with their conjugates and has the following form^70,71^

27 $$\begin{aligned} dm = TdS + VdP + \phi dg, \end{aligned}$$

where *m*(*S*, *P*, *g*) is a black hole mass function with the entropy *S*, pressure *P*, and charge parameter *g* as dynamical variables. *T* is the black hole temperature, *S* is the entropy, and *V* is the thermodynamic volume. The conjugate variables of *S*, *P*, and *g* are the temperature *T*, the thermodynamical volume *V*, and the potential $\phi$. The modified Smarr formulae turn out to be

28 $$\begin{aligned} m = 2TS' - 2PV +{\phi }g . \end{aligned}$$

The entropy $S'$ resulting from (28) reads

29 $$\begin{aligned} S' = \frac{\pi (g + x_+)^3}{x_+}, \end{aligned}$$

The entropy $S'$ is different from the entropy in Eq. (24), which satisfies the first law of thermodynamics. This discrepancy raises concerns about the consistency of the Smarr formula. In other words, the first law does not agree with the Smarr formula for the regular AdS black hole at distances where *g* is comparable with the event horizon radius unless we are working in the large-distance domain ($x_+\gg g$), where corrections of *g* can be neglected. The two entropies coincide, satisfying the standard area law, $S'=S=\pi x_{+}^2$.

The equation of state $P = P(V,T)$ for the regular AdS black hole is found by combining the expressions for the temperature *T*, volume *V*, and pressure *P* such that

30 $$\begin{aligned} P = \frac{2g(2+2 \pi T\zeta _v)+(-1+2\pi T\zeta _v)\zeta _v}{2 \pi \zeta _v{^3}}, ~~~~~~~ \zeta _v =\Big [-2g+\Big (\frac{6V}{\pi }\Big )^\frac{1}{3}\Big ]. \end{aligned}$$

The thermodynamic stability of the black hole requires investigation of the Gibbs free energy behaviour^24,51^. We are interested in the regions with negative free energy and identify where black holes are thermally favoured over the reference background. The free energy of the black hole is calculated from^51^,

31 $$\begin{aligned} G_+ = m_+ - T_+S_+, \end{aligned}$$

On using expressions $m_+$, $T_+$ and $S_+$, respectively from Eqs. (20), (21) and (24), we obtain

32 $$\begin{aligned} G_+= & {} \frac{1}{2{x_+}^2(g+x_+)}\Big [(g+x_+)^4(1+{x_+}^2)-\frac{1}{2}({3x_+}^3 + (-2g+x_+)(-2g^3 + 6g{x_+}^3 + {x_+}^3 + 6g{2x_+}\log [x_+]))\Big ], \nonumber \\ \end{aligned}$$

which reduces to Gibb’s free energy of Schwarzschild-AdS black holes^72,73^, when $g=0$,

33 $$\begin{aligned} G_+ = \frac{{x_+}^4(1+{x_+}^2)-\frac{1}{2}{x_+}^3({x_+}+3{x_+}^3)}{2{x_+}^3}. \end{aligned}$$

**Figure 7:**
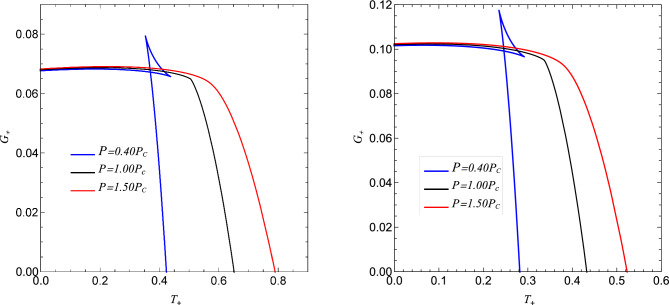
The plot of Gibbs free energy $G_+$ vs temperature $T_+$ exhibiting a liquid-gas phase transition for $P< P_c$ and no transition for $P> P_c$ obtained for a particular value of *g* and corresponding critical pressure $P_c$ with $P_c = 0.612765$ and $P_c=0.27234$, respectively, for $g = 0.02$ (left) and $g=0.03$ (right).

**Figure 8:**
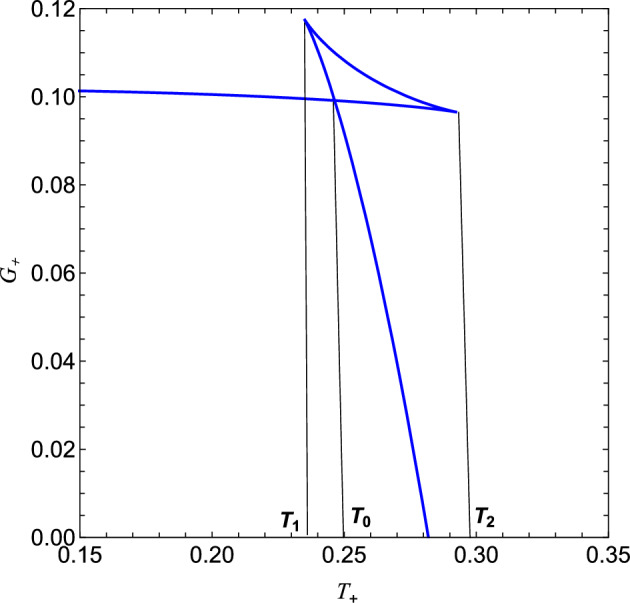
The generic plot of Gibbs free energy $G_+$ vs horizon $T_+$ for $P < P_c$.

Since the global stability of the system is measured by Gibbs free energy, its global minimum is estimated to be the preferred state of the black hole^74^. We have presented the behaviour of Gibbs free energy $G_+$ vs horizon $x_+$ and temperature $T_+$ of regular AdS black hole in (cf. Fig. 6) and (cf. Fig. 7). We know that black holes with negative Gibbs free energy are globally stable; hence, from (cf. Fig. 6), one can clearly say that large black holes are globally stable, whereas black holes with small horizon radii are not stable globally. (cf. Fig. 6), shows the behaviour of Gibbs free energy $G_+$ vs temperature $T_+$, For the values of pressure below the critical pressure $P_c$, the *G* - *T* diagram exhibits a swallowtail structure^75^ where the Gibbs free energy of the black hole intersects with itself, which is indicative of first-order phase transition between small black hole and large black hole (cf. Fig. 7). By analyzing the Gibbs free energy, one can observe that at a pressure lower than the critical value ($P < P_c$), the Gibbs free energy exhibits a characteristic swallowtail behaviour, implying that small black hole /large black hole first-order phase transition occurs. Indeed, for $P < P_c$, the $G - T$ graph exhibits three black hole states: Stable small black hole, Unstable intermediate black hole and stable large black hole (cf. Fig. 8). Further, the transition of the small black hole /intermediate black hole occurs at the inflexion point $T_2$, and the inner meet is at $T_1$. Thus, is the preferred state when *T* $\epsilon$ $[T_0, T_2]$, and for $T < T_0$, the preferred state is stable small black hole. Thus, the small black hole undergoes a first-order phase transition to a large black hole at $T = T_0$ (cf. Fig. 8). The second order phase transition is shown through the discontinuity of specific heat and the cusp in the $G - T$ plot when we take $P = P_C$^76^. We see no swallowtail structure above the critical pressure $P_c$, suggesting that no first-order phase transition is occurring. We also find that Gibbs’s free energy changes its sign, which signifies a Hawking-Page phase transition between the thermal radiation and black hole^5^. After discussing the global thermodynamical stability of regular-AdS black hole, we next turn to the local stability or thermal stability. The reason to consider local stability is that even when a black hole configuration is globally stable and can be locally unstable^77,78^. One can investigate the thermodynamic stability from the Specific heat $C_V$ and $C_P$ of the system^10^, where $C_V$ is heat capacity at constant volume and $C_p$ is heat capacity at constant pressure. $C_V= {T} \Big (\frac{\partial S}{\partial T}\Big )_{V,g} = 0$ which turns out to be zero in our case and one with constant pressure $C_p = {T} \Big (\frac{\partial S}{\partial T}\Big )_{P,g}$ which yields

34 $$\begin{aligned} C_p^+ = \frac{2\pi (g+x_+)^4(3{x_+}^2-2g+x_+)}{3{x_+}^4(2g+x_+)+{x_+}(2g^2+4g{x_+}-{x_+}^2)}, \end{aligned}$$

**Figure 9:**
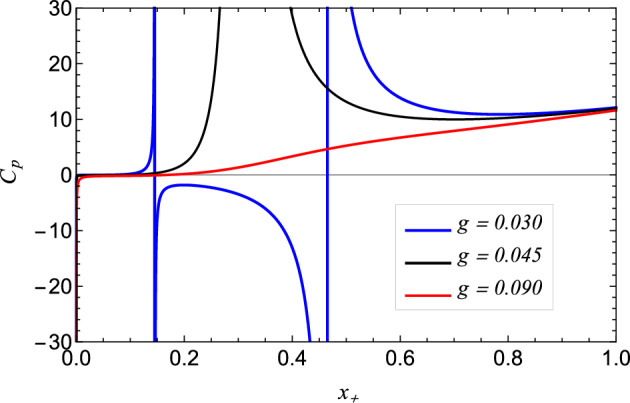
The plot of specific heat $C_p$ vs $x_+$ for $g = 0.03$ (blue curve), $g = g_c \approx$ 0.04531 (black curve) and for $g = 0.09$ (red curve).

Let’s examine the black hole’s thermodynamic stability and potential phase transitions by evaluating its heat capacity at constant pressure $C_P^+$. We can deem locally a black hole thermodynamically stable if its heat capacity $C_P^+>0$ is positive. Conversely, when $C_P^+<0$, the black hole is in a state of thermodynamic instability. We plot $C_P^+$ in (cf. Fig. 9). When $g = 0.03$ < $g_c$, there are three branches divided by two asymptotes at $x_+ \cong {0.144}$ and $x_+$ $\cong {0.4643}$. Small black holes with $x_+$ $\lesssim 0.144$ and Large black holes with $x_+$ $\gtrsim 0.471$, for which ($C_p^+ >0$) signifying their local thermodynamical stability and the intermediate black holes with $0.144 \lesssim x_+ \lesssim 0.471$ having $C_p^+<0$ are thermodynamically unstable. The specific heat suffers a discontinuity at two points analogous to the maximum and minimum temperature (cf. Fig. 7), confirming the existence of phase transition. When $g = g_c = 0.045314421$, we have two branches corresponding to small and large thermodynamically stable black holes, which coexist at the inflexion point $x_+ = 0.311445$ and for $g = 0.09 > g_c$, only thermodynamically stable black hole exit.

**Figure 10:**
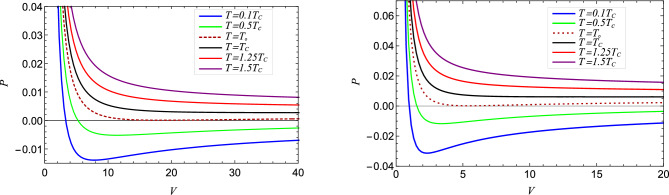
The isotherms of black holes in *P*-*V* plane for two different values of parameter $g = 0.3$ with $T_c = 0.03369221$ (left) Solid black curve and $g = 0.2$ with $T_c = 0.05053832076$ (right) Solid black curve. The temperature of isotherms decreases from top to bottom.

The $P - V$ diagram depicted in (cf. Fig. 10) plots the various isotherms of the equation of state (30). For values of temperature *T* higher than the critical temperature $T_c$, the plot shows the isotherms follow ideal gas behaviour. The isotherms undergo liquid-gas like phase transition^39,79^ for $T < T_c$ governed by the Maxwell’s equal area law^80^ and have a point of inflection at $T_c$, which can be obtained by using

35 $$\begin{aligned} \bigg (\frac{\partial P }{\partial x_+}\Bigg )_{T} = 0 = \bigg (\frac{\partial ^2 P }{\partial x_+^2}\Bigg )_{T} . \end{aligned}$$

The high-pressure regime is associated with small black holes, while the low-pressure regime corresponds to large black holes. An oscillating branch emerges between the small and large black holes, characterized by a van der Waals first-order phase transition. During this transition, the distinction between small and large black holes becomes indistinct as the black holes shift between the two categories. The Eq. (35) yields the expression for the critical temperature $T_c, P_c$ and $V_c$

36 $$\begin{aligned} P_c \simeq \frac{0.000245}{g^2},\quad V_c \simeq 2044.12 g^3, \quad T_c \simeq \frac{0.0101077}{g} . \end{aligned}$$

The point at which pressure, volume and temperature have the critical values as given in Eq. (36), known as the critical point, is the point at which we cannot distinguish small and large black holes (small and large black holes coexist). The universal constant for a regular black hole is obtained as

37 $$\begin{aligned} \epsilon = \frac{ P_c V_c^{\frac{1}{3}}}{T_c} \approx 0.31 , \end{aligned}$$

which is slightly smaller than the value of 3/8 of the van der Waals gas.

## Critical exponents

Lastly, we assess the critical exponents that describe specific heat, parameter order, isothermal compressibility, and the critical isotherm, viz., $\alpha$, $\beta$, $\gamma$ and $\delta$, which elucidate the characteristics of physical properties in proximity to the critical point^11^. We focus on the numerical computation of these critical exponents for the black hole system. It proves advantageous to define the reduced thermodynamic temperature, volume, and pressure as follows^81^:

38 $$\begin{aligned} p = \frac{P}{P_c}, \quad v = \frac{V}{V_c}, \quad \tau = \frac{T}{T_C}. \end{aligned}$$

In calculating the critical exponents, our initial step involves deriving the relevant equation of state. This is achieved through the substitution of the introduced reduced parameters We derive the law of corresponding states by plugging in the aforementioned expressions Eq. (38) into the equation of state (Equation 30):

39 $$\begin{aligned} p = \frac{0.997951 + v^{1/3} (-2.61895 - 0.33265\tau )+2.61895v^{2/3} \tau }{(-0.127017 +v^{1/3})^3} . \end{aligned}$$

The aforementioned law holds universally and remains valid under broader assumptions than those stated in Eq. (30). To further elaborate, we can extend our analysis by considering the quantities

40 $$\begin{aligned} t = \tau -1 , \quad \omega = v-1. \end{aligned}$$

we can approximate (39) as

41 $$\begin{aligned} p \simeq 1 + 3.43649t - 1.47883 \omega t+0.0556696 \omega ^3 +{\mathscr {O}} (wt^2), \end{aligned}$$

The critical exponents can be found through the relations:

42 $$\begin{aligned} C_V= & {} T\frac{\partial S}{\partial T}\bigg \vert _V \propto {|t|}^{-\alpha }, \end{aligned}$$

43 $$\begin{aligned} \eta= & {} V_l - V_S \propto {|t|}^{\beta }, \end{aligned}$$

44 $$\begin{aligned} \kappa _T= & {} -\frac{1}{V}\frac{\partial V}{\partial P}\bigg \vert _V \propto {|t|}^{-\gamma }, \end{aligned}$$

45 $$\begin{aligned}{} & {} |P - P_c|_{T-T_c} \propto {|V-V_c|^\delta }, \end{aligned}$$

In this context, $V_s$ and $V_l$ represent the volumes of the small and large black holes, respectively, while $\kappa _T$ denotes the compressibility coefficient. The initial exponent, denoted as $\alpha$, characterizes the response of specific heat at constant volume. In our particular scenario, it assumes a value of zero, i.e., $\alpha = 0$, since $C_V = 0$, indicating no sensitivity to temperature variations (*t*).

The second exponent, $\beta$, can be evaluated using (41) along with the application of Maxwell’s area law.

46 $$\begin{aligned} \oint VdP = 0. \end{aligned}$$

This involves substituting the oscillatory component of the isotherm with an isobar in a canonical ensemble. This substitution implies that, during the transition, the system’s pressure remains constant instead of undergoing oscillations. The differentiation of Eq. (41) with respect to a fixed *t* yields:

47 $$\begin{aligned} dp = -(1.4788t+ 0.16701 \omega ^2)d\omega , \end{aligned}$$

From (41), (46) and (47) we get the following system:

48 $$\begin{aligned} 0 = \int _{\omega _s}^{\omega _l}(1.4788t+ 0.16701 \omega ^2)d\omega , \end{aligned}$$

49 $$\begin{aligned} p= t (3.43 - 1.4788\omega _l)- 0.05567\omega _l^3-t(3.43- 1.4788\omega _s)+0.05567\omega _s^2. \end{aligned}$$

The solutions of the above two equations are

50 $$\begin{aligned} \omega _l = - \omega _s, \quad \omega _l =- \omega _s \simeq 5.154\sqrt{-t}, \end{aligned}$$

Thus Eq. (43) can be written a

51 $$\begin{aligned} \eta = V_l - V_S = V_c(\omega _l-\omega _c) \simeq 10.31V_c\sqrt{-t}, \end{aligned}$$

identifying the exponent $\beta$ with the value $\beta$ = 1/2. For the third exponent $\gamma$, we substitute (38), (40) and (47) into the compressibility (44), retrieving

52 $$\begin{aligned} \kappa _T = - \frac{1}{(\omega +1)P_c}\frac{d\omega }{dp} \propto {\frac{1}{t}}, \end{aligned}$$

Therefore, the exponent $\gamma$ equals to unity, $\gamma = 1$. For the fourth exponent $\delta$, we set $T = T_c$ having that way t = 0

53 $$\begin{aligned} p-1 = 1-0.05567\omega ^3. \end{aligned}$$

Upon equating the exponent $\delta$ to the specific value $\delta = 3$, it becomes evident that the critical exponents of the Regular-Ads black hole ($\alpha$, $\beta$, $\gamma$, $\delta$) = (0, 1/2, 1, 3) coincide with those of a van der Waals gas. Therefore, the critical aspects associated with black holes play a crucial role in exploring critical behaviour, phase transitions, and critical exponents.

## Concluding remarks

Kastor et al.^34^ initiated the field of extended phase space thermodynamics and black hole chemistry and subsequently developed by many researchers. One significant advancement in this area involves considering the negative cosmological constant as the pressure and introducing a new pair of thermodynamic variables: the thermodynamic volume as a conjugate variable. However, this change requires reinterpreting the mass parameter as the enthalpy. Through this formalism, researchers have discovered diverse thermodynamic behaviours exhibited by various black holes across multiple gravity models. Notably, extensive studies have been conducted on phase transitions resembling those observed in the liquid-gas system of van der Waals, as well as some exotic types. Despite the well-established thermodynamic connection to van der Waals fluids, numerous unresolved problems and unanswered questions still need to be solved in this field. Therefore, observing these phenomena in more complex scenarios involving regular black holes coupled with NED would be intriguing. With this motivation, we analysed the extended phase space thermodynamics for a class of physically reasonable^61^ regular black holes in an AdS background. From a geometric standpoint, we found a Lagrangian to derive an exact solution for static spherically symmetric regular AdS black holes, encompassing Schwarzschild AdS black holes when the NED switched off ($g \rightarrow 0$). While subject to parameter constraints, these derived-AdS black holes can possess two horizons, describing a range of charged, self-gravitating objects. It includes an extremal black hole with degenerate horizons and a non-extremal black hole with Cauchy and event horizons.

After calculating the desired thermodynamic variables, we analysed several quantities, including the Hawking temperature, entropy, specific heat at constant pressure, and Gibbs free energy in the extended phase space. The resulting expressions for specific heat and Gibbs free energy unveiled a first-order phase transition between small and large black holes. This transition is apparent in the $P-V$ diagram, where, below the critical Temperature $T_c$, the regular-AdS black hole exhibits a phase transition akin to the coexistence of liquid and gas phases. We also explored the $P-V$ criticality of our regular-AdS black hole, and by examining the behaviour of various thermodynamic quantities near the critical point^79,82^, we determined the values of the critical exponents. We have also computed the critical exponents of the phase transition and found that the thermodynamic exponents coincide with those of the van der Waals fluid.

Thus, we have investigated extended-phase space thermodynamics for a class of regular black holes in the AdS background. We have derived the first law and a Smarr-like formula for static and spherically symmetric regular black holes. Our analysis will yield significant insights into extended-phase space thermodynamics.

## Data Availability

All data generated or analysed during this study are included in this published article.
